# Embracing complexity: making sense of diet, nutrition, obesity and type 2 diabetes

**DOI:** 10.1007/s00125-023-05873-z

**Published:** 2023-02-14

**Authors:** Nita G. Forouhi

**Affiliations:** MRC Epidemiology Unit, University of Cambridge School of Clinical Medicine, Institute of Metabolic Science, Cambridge Biomedical Campus, Cambridge, UK

**Keywords:** Diet, Epidemiology, Nutrients, Nutrition, Obesity, Quality, Quantity, Review, Study design, Type 2 diabetes

## Abstract

Nutrition therapy has been emphasised for decades for people with type 2 diabetes, and the vital importance of diet and nutrition is now also recognised for type 2 diabetes prevention. However, the complexity of diet and mixed messages on what is unhealthy, healthy or optimal have led to confusion among people with diabetes and their physicians as well as the general public. What should people eat for the prevention, management and remission of type 2 diabetes? Recently, progress has been made in research evidence that has advanced our understanding in several areas of past uncertainty. This article examines some of these issues, focusing on the role of diet in weight management and in the prevention and management of type 2 diabetes. It considers nutritional strategies including low-energy, low-fat and low-carbohydrate diets, discusses inter-relationships between nutrients, foods and dietary patterns, and examines aspects of quantity and quality together with new developments, challenges and future directions.

**Graphical abstract:**

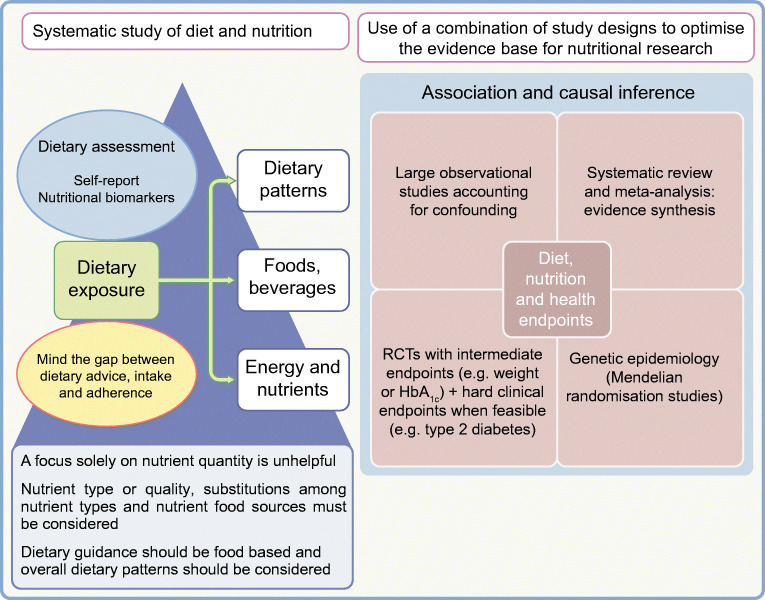

**Supplementary Information:**

The online version contains a slide of the figure for download available at 10.1007/s00125-023-05873-z.

## Diet, nutrition and type 2 diabetes: what is the evidence?

Diabetes is a metabolic disorder with the potential for multiple adverse health consequences. It is also a public health challenge, with a rising global burden. Estimates indicate that there were approximately 537 million people worldwide with diabetes in 2021, which is projected to rise to 783 million by 2045, with type 2 diabetes constituting the majority (>90%) of this burden [1]. Diet and nutrition are of indisputable significance in reducing this burden because the development of type 2 diabetes is characterised by obesity and insulin resistance, leading to hyperglycaemia, and both weight and glycaemic control are directly related to food consumption.

Diet and nutrition are thus central as modifiable factors in both the management and the prevention of type 2 diabetes. This is supported by three lines of evidence. First, when adhered to, medical nutrition therapy in those with type 2 diabetes can match or exceed the glycaemic control that can be achieved by glucose-lowering medication in the short term, and can be useful in maintaining control [2]. Second, the proof of principle was established in the early 2000s that, among people with non-diabetic hyperglycaemia, the onset of type 2 diabetes can be delayed or prevented, with as much as a 58% relative risk reduction, through a supported intensive lifestyle intervention including dietary changes and physical activity [3]. The real-world impact of lifestyle modification strategies has been demonstrated [4], outside the highly controlled conditions of clinical trials, and such a strategy has been found to be effective in the UK National Health Service (NHS) [5]. Third, it has been demonstrated that remission of type 2 diabetes can be achieved through dietary means [6], resulting in a major shift in scientific understanding of the pathophysiology of type 2 diabetes, from a condition previously thought to be progressive and irreversible to one that can be brought under control to normal functioning.

**Fig. 1:**
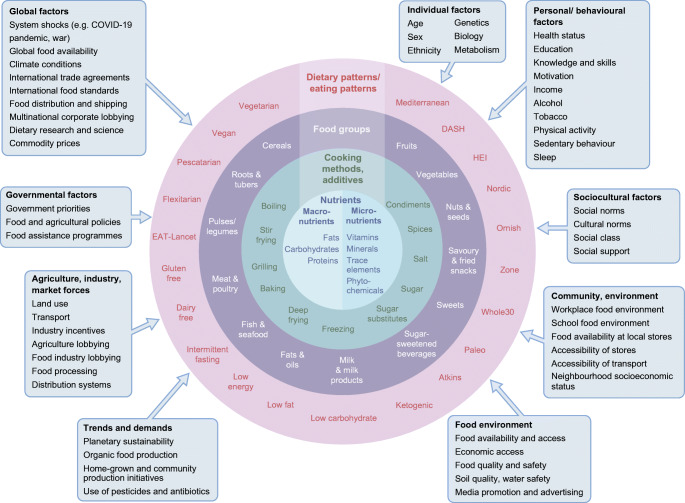
Complexity of diets and multiple influences affecting food intakes. HEI, Healthy Eating Index. Influencing factors (boxes) adapted with permission from Afshin et al [83] © 2014 John Wiley & Sons. This figure is available as a downloadable slide.

However, defining the optimal diet for type 2 diabetes is a challenge and dietary strategies used in research have varied between different studies. This is largely because diet is intensely complex, with multiple components and influences on food consumption (Fig. 1). Concomitantly, interest in diet, nutrition and health is intense, with a deluge of scientific publications, matched equally by popular media coverage that is saturated with nutrition over-claims and ‘miracle diets’. This is also a field where vested interests are rife [7]. A search on PubMed (25 November 2022) using the terms ‘diet OR nutrition OR food OR nutrient OR dietary pattern OR diet quality’ and ‘type 2 diabetes OR non-insulin dependent diabetes’ yielded 52,833 hits, with over 3000 articles published each year since 2014; repeating the search using the term ‘obesity’ yielded 165,617 hits. What evidence should we trust?

The hierarchy of evidence framework and quality assessment tools have been applied to sift through the vast amount of evidence. Several reviews of the research evidence have been carried out [8–14], enabling the incorporation of the best available evidence in dietary guidelines issued by authoritative agencies, including but not limited to the ADA [15] and Diabetes UK [16].

In a nutshell, this evidence highlights some key dietary principles. Healthy weight maintenance is critical to both prevent and manage type 2 diabetes; a pattern of food intake that mitigates type 2 diabetes risk includes the habitual consumption of vegetables, fruits, legumes, whole grains and cereal fibre, dairy products such as yoghurt, and nuts, and several overall dietary patterns are effective. In contrast, type 2 diabetes risk is elevated with a pattern of habitual dietary intake that includes processed and unprocessed red meat, refined grains and sugar-sweetened beverages. This evidence provides support that some foods should be emphasised and promoted while the consumption of others should be reduced or avoided, rather than the adage about everything in moderation.

This article does not cover the wide range of topics already discussed in existing reviews and guidelines. It focuses instead on selected hot topics that have been the subject of debate and on new developments in understanding in the field.

## Weight management at the core, but how?

Body weight with increased adiposity is mechanistically linked to both the development and the progression of type 2 diabetes, typified by resistance to insulin action (insulin resistance) and an inadequate compensatory insulin secretory response by pancreatic beta cells. The relationship between adiposity, insulin resistance and beta cell function varies between individuals but the benefits of weight loss apply across the different pathophysiologies [17]. Weight loss is related to improved glycaemic control: the greater the weight loss, the greater the improvement in HbA_1c_. A weight loss goal of 5–7% of initial body weight for people with overweight or obesity is recommended for clinical benefit, while weight loss of 15% can be disease modifying with the possibility of remission of type 2 diabetes [2, 18].

Of the three options for weight management, bariatric surgery and pharmacotherapy are effective, but dietary strategies offer population-wide benefits without medicalisation. However, the weight loss and weight management diet market is vast and is projected to increase from US$192.2 billion in 2019 to US$295.3 billion by 2027. This promotion of a vast range of dietary products and strategies can be bewildering. An important question is therefore which dietary strategies are effective?

## Remission of type 2 diabetes through diet-related weight loss

The proof of principle of the potential for reversibility or remission of type 2 diabetes with weight loss came first from the field of bariatric surgery [19, 20]. However, surgery is not suitable for, or acceptable to, all people with type 2 diabetes. Surgery also has the potential for complications, side effects and challenges. One such challenge is the large prevalence of type 2 diabetes, which renders surgery an unrealistic option at the scale required, even if it were financially possible. There is high interest, therefore, in dietary means to achieve diabetes remission.

The nutritional basis for the remission of type 2 diabetes used in the UK-based Diabetes Remission Clinical Trial (DiRECT) was centred on major caloric restriction and weight loss with an associated reduction in hepatic fat and hepatic glucose output and improved beta cell function [6]. Among people with type 2 diabetes in primary care who were randomised to either a diet very low in energy (very low calorie diet) or usual care, mean body weight fell by 10 kg in the intervention group and 46% remained free of diabetes (i.e. in remission; HbA_1c_ <48 mmol/mol [<6.5%]) at 1 year and off all glucose-lowering and antihypertensive medications [21]. The intervention comprised total diet replacement (3452–3569 kJ/day [825–853 kcal/day] liquid formula diet for 12–20 weeks), stepped food reintroduction (2–8 weeks) and then structured support for weight loss maintenance. The greater the weight loss, the greater the likelihood of remission (86% at 1 year for weight loss ≥15kg; 57%, 34% and 7% for weight loss of 10–15 kg, 5–10 kg and <5 kg respectively). In addition, the effects were durable, with 36% of people in sustained remission at 2 years [22]. Further research is needed to understand the longer term effects of remission on the complications of type 2 diabetes, but current results support the remission of type 2 diabetes as a practical target in primary care.

In an endorsement of this approach, the UK NHS has rolled out a 12 week intervention consisting of a low-energy meal replacement diet for people with type 2 diabetes and a BMI >27 kg/m^2^ (or >25 kg/m^2^ if from a minority ethnic group in whom risk occurs at a lower BMI) (https://www.england.nhs.uk/2022/01/nhs-soups-and-shakes-diet-helps-thousands-shed-the-pounds/). The goal is to recruit 5000 people from general practice; over 2000 people have already participated, showing the feasibility of this approach.

## A focus on nutrients for weight and glycaemic control

Traditionally, dietary guidance has focused on macronutrient composition. Most dietary guidelines recommend intakes of <30–35% of energy from total fat, 45–55% of energy from carbohydrates and the remainder, ~15–20% of energy, from protein, both in the general population and in those with type 2 diabetes. For weight management, low-fat diets were favoured based on the higher energy density of fat, at 38kJ/g (9 kcal/g), compared with that of carbohydrate or protein, at 17kJ/g (4 kcal/g). More recently, low-carbohydrate diets have gained popularity. The optimal macronutrient composition is hotly debated.

### Low-fat or low-carbohydrate diets for weight management?

The Look-AHEAD: Action for Health in Diabetes (Look-AHEAD) trial compared an intensive lifestyle intervention with a control condition of support and education in people with type 2 diabetes. The weight loss strategy, comprising energy reduction (5021–7531 kJ/day [1200–1800 kcal/day]) through a low-fat diet, was effective. Greater weight loss was achieved in the intervention group at 1 year, with a net difference in weight of –7.9% (95% CI –8.3% to –7.6%); at year 4, the net difference in weight was –3.9% (95% CI –4.4% to –3.5%) [23]. Similar low-fat diet approaches have been used in other trials of the primary prevention of type 2 diabetes [3]. In contrast, in the energy-deficit diet in the type 2 diabetes remission trial (DiRECT), the proportions of macronutrients were inconsequential, with >50% of energy coming from carbohydrates [22]. A recent umbrella review of the evidence concluded that weight management in type 2 diabetes using hypocaloric diets does not depend on any particular macronutrient profile [24].

More broadly, among adults with overweight or obesity in the population without consideration of type 2 diabetes, individual studies show differing results favouring one nutrient or another but, when the totality of the evidence is appraised, both low-fat and low-carbohydrate diets of varying protein content are effective for weight loss [25]. The challenge lies in adherence to the prescribed diets. A systematic review of the effects of low-fat and low-carbohydrate diets on weight loss in RCTs of at least 1 year’s duration and with a similar intervention intensity across groups found that low-fat diets were efficacious compared with usual intake [26]. But, when low-fat diets were compared with low-carbohydrate diets, there was greater weight loss in the low-carbohydrate diet group. However, the magnitude of the difference in weight loss between low-carbohydrate and low-fat diets was modest at only 1.15 kg, which is statistically significant but may have little clinical meaning. As a limitation, caloric restriction was a component of many of the weight loss interventions included, but not all; for example, some included studies gave dietary advice to eat a low-carbohydrate diet ad libitum [26]. Future research should seek to address design limitations; however, current research indicates that small effects on weight loss from one macronutrient type or another are unlikely to be of clinical significance. A key challenge is weight maintenance and prevention of weight regain, which is typical following weight loss.

Although overall dietary carbohydrate or fat content has been extensively studied in relation to weight loss and maintenance, protein intake has been less so. Higher protein intake after weight loss has been shown to result in significantly lower weight regain, related to increased satiety and energy efficiency [27]. For early weight loss maintenance over 6 months, an RCT tested different combinations of protein consumption and glycaemic index (GI) compared with a control diet among those who had lost at least 8% (equivalent to 11 kg) of their initial weight on a 3347 kJ/day (800 kcal/day) diet [28]. Consuming a low-protein/high GI diet led to subsequent weight regain (mean of 1.7 kg [95% CI 0.5 to 2.9]), while a modest increase in protein content and a modest reduction in GI led to improvements (reductions) in the degree of weight regain over 6 months. Evidence for long-term weight loss maintenance is generally sparse. Observational prospective data from the National Weight Loss Registry indicated that weight loss maintenance over 10 years was related to low-fat-based energy restraint combined with physical activity [29]. Further research is needed to better understand the dietary strategies and other factors important in weight loss maintenance.

### Low-carbohydrate diets for glycaemic control in type 2 diabetes

For glycaemic control in type 2 diabetes, studies from clinical practice or from digital or commercial programmes have promoted low-carbohydrate diets based on significant benefits for HbA_1c_, of a mean decrease of 11 mmol/mol (1% unit decrease), together with reductions in glucose-lowering medication use [30, 31]. Interpretive challenges include the presence of bias owing to the lack of randomisation, self-selection into groups and unbalanced sample sizes or intensities of interventions in the study arms and lack of a comparator group. However, a number of systematic reviews and meta-analyses of RCTs are available that reduce such limitations [32–39].

Evidence from RCTs indicates that lower carbohydrate diets have benefits over higher carbohydrate diets in the short term up to 6 months, but these are not maintained over time [34, 36]. In the UK, the Scientific Advisory Committee on Nutrition appraised the available evidence, including 48 individual RCTs from eight systematic reviews. It concluded that lower carbohydrate diets were effective for glycaemic control in type 2 diabetes compared with higher carbohydrate diets, with a greater reduction in HbA_1c_ (weighted mean difference –4.7 mmol/mol [–0.47%]) in the short term (3–6 months), but this benefit was not maintained at 12 months [39].

Despite extensive research on low-carbohydrate diets, there are several challenges that limit firm conclusions. First, definitions of what a ‘low-carbohydrate diet’ is range from moderate carbohydrate restriction to very-low-carbohydrate or ketogenic diets (see Text box ‘Definitions of carbohydrate-focused diets’). Across RCTs, prescribed carbohydrate intakes in the lower carbohydrate groups ranged widely, from 14% to 50% of energy intake, and reported carbohydrate intakes were moderate at 26–45% of energy intake in the majority of the primary RCTs [39]. Second, in the case of isoenergetic diets (maintaining the same overall energy intake), a low-carbohydrate diet is by default higher in fat and vice versa. As many individual studies did not specify isoenergetic study arms, it is difficult to tease out whether the glycaemic change was influenced by differential changes in weight as a result of differing energy intakes. Third, because of differences in or a lack of information in study protocols on adjustment of glucose-lowering medication, it is hard to infer whether criteria for remission of type 2 diabetes were met [40]. 
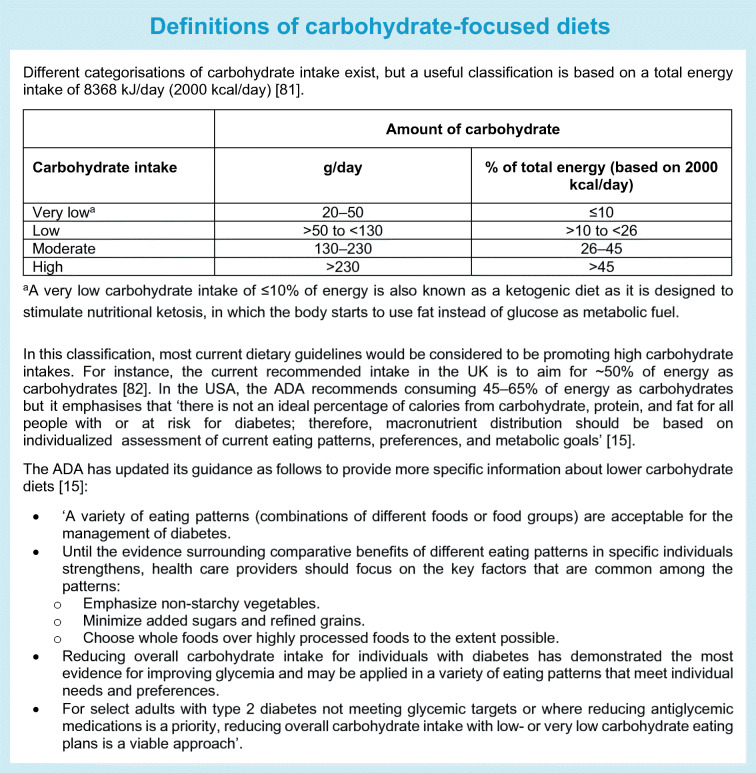


Low-carbohydrate diets seem to be generally safe and well tolerated in the short term; concerns in the longer term relate to the potential atherogenic lipid profile [38, 41] or micronutrient deficiency [42] or their use in people with chronic kidney disease or pregnant women, in whom there is a need for further evaluation. Accumulating evidence from prospective studies with long-term follow-up data indicates that both high and low intakes of carbohydrates may have adverse health impacts on mortality risk, with a U-shaped relationship [43]. However, such research has been carried out in general populations and needs to be replicated, and further research is needed in those with type 2 diabetes. In the meantime, the ADA dietary guidelines for people with diabetes were updated in 2019, making it explicit that low-carbohydrate diets can be endorsed (see Text box ‘Definitions of carbohydrate-focused diets’).

## Nutrition and pathways to obesity and type 2 diabetes

The above focus on energy and macronutrients is rooted in two contesting mechanistic explanations that link dietary intake to obesity and type 2 diabetes. In the energy balance model, energy matters because the law of thermodynamics dictates that when energy intake exceeds energy expenditure weight gain occurs. The link between obesity and the development of type 2 diabetes is strong and, with caloric deficit-induced weight loss, remission of type 2 diabetes is possible. In these scenarios, a calorie is a calorie and excess calories result in adipose tissue accumulation and weight gain.

In contrast, the ‘carbohydrate–insulin model’ proposes that obesity is a cause, not the consequence, of excess caloric intake [44]. Here, the dysregulation of fat storage and metabolism is the central defect, driven by high-carbohydrate diets that produce spikes of hyperinsulinaemia that promote glucose uptake into tissues, suppress release of fatty acids from adipose tissue and stimulate fat and glycogen storage. Thus, less energy remains available for use by the rest of the body, driving hunger and overeating. In this scenario, not all calories are equal. It has been proposed that energy from refined carbohydrates promote a disturbed hormonal milieu linked with increased hunger, a slower metabolic rate and reduced energy expenditure, leading to adiposity.

The debate between these mechanistic processes continues [45–47]. However, it is increasingly clear that a focus on energy intake does not account for the impact that diet quality has on long-term weight gain and type 2 diabetes through diverse physiological processes. These include diet-induced thermogenesis, brain reward, appetite, hunger, satiety, digestion, the release and action of hormones, for example insulin, hepatic de novo lipogenesis, interactions with the gut microbiome and energy expenditure [48]. Moreover, a focus on considering a single macronutrient type has limitations that can lead to unhelpful reductionist messages to avoid a macronutrient without reference to its quality and food sources.

## Beyond a focus on nutrient quantity: the relevance of nutrient type, quality and food sources

RCTs of macronutrient manipulation have focused exclusively on quantity. This ignores the fact that health effects will vary substantially by nutrient type or quality. For dietary fats, a vast literature exists on the importance of distinguishing between saturated, polyunsaturated, monounsaturated and trans fats. Health effects also vary by carbohydrate type (starch, sugar or fibre), degree of processing (whole grain vs refined grain), glycaemic response after consumption (GI and load) and food structure (solid or liquid form).

There is substantial evidence from meta-analyses for inverse (beneficial) associations between the consumption of fibre [49], particularly cereal fibre [50] and wholegrains [11], and the incidence of type 2 diabetes. However, evidence is more mixed for the dietary GI, which reflects the differential blood glucose-raising potential of foods with similar carbohydrate content, and a related measure, the glycaemic load (GL), which accounts for the amount of available carbohydrate. For example, the meta-analysis by Reynolds et al found inverse associations between fibre intake and several disease endpoints, including type 2 diabetes and mortality, but associations with GI and GL were non-significant [49]. Mixed and inconclusive results were also reported in reviews of a link between GI, GL and HbA_1c_ or fasting glucose [15]. The OmniCarb RCT compared four diets with varying GI and carbohydrate content in overweight or obese individuals with hypertension or pre-hypertension. This was a crossover feeding study with each diet based on a Dietary Approaches to Stop Hypertension (DASH)-type diet pattern [51]. Compared with a high GI (65% on the glucose scale), high-carbohydrate (58% energy) diet, a low GI (40% on the glucose scale), low-carbohydrate (40% energy) diet did not significantly improve insulin sensitivity, lipid levels or blood pressure. This type of evidence indicates that GI values have a low utility, but further research contradicts this. Other reviews with a more nuanced approach have reported a positive association between GI or GL and type 2 diabetes [52]. Similarly, some reviews and individual large cohorts have also reported a positive (adverse) association of high GI or GL with CHD or CVD [53], as well as a likely benefit of low GI or GL dietary patterns for glycaemic control and cardiometabolic risk factors in people with type 1 diabetes or type 2 diabetes [54]. A take-home message is that multiple aspects of carbohydrate quality are relevant and should be considered where possible because intakes of fibre, wholegrain and the GI and GL values of foods are likely to be highly correlated and may have confounding effects if not accounted for in diet–disease associations.

A point to note is that, when consumption of one nutrient type is manipulated (to eat less or more of it), this impacts the consumption of other nutrient types—the so-called ‘nutrient substitution’, in which one nutrient substitutes for another within isoenergetic consumption. Moreover, there are both ‘healthy’ and ‘unhealthy’ low-fat or low-carbohydrate diets.

The Diet Intervention Examining The Factors Interacting with Treatment Success (DIETFITS) RCT tested diet quality, comparing ‘healthy’ low-carbohydrate and low-fat regimens [55]. Both diet groups were instructed to maximise their non-starchy vegetable intake, minimise added sugars, refined flours and trans fats and focus on whole foods. Both diet types were effective, with a mean weight loss of 5.3 kg and 6 kg for the healthy low-fat and healthy low-carbohydrate diets, respectively, at 12 months, but there was no significant between-group difference [55]. In both diet groups there were also improvements at 12 months in secondary outcomes, including fasting glucose and insulin levels, body fat percentage, waist circumference, blood pressure and lipid profiles, except for LDL-cholesterol level, which was reduced in the low-fat group but increased in the low-carbohydrate group.

A crossover trial compared different levels of carbohydrate restriction and food sources in people with prediabetes or type 2 diabetes over two 12 week periods. Carbohydrates comprised <20% of energy in the very-low-carbohydrate ketogenic diet and <40% in the low-carbohydrate Mediterranean-style diet [56]. Both diets incorporated non-starchy vegetables and avoided added sugars and refined grains; the ketogenic diet avoided legumes, most fruits (except a few berries in small amounts) and whole grains whereas the Mediterranean-style diet incorporated these foods. Both diets resulted in improvements that were not significantly different. Specifically, mean HbA_1c_ levels decreased by 9% and 7% in the ketogenic and Mediterranean-style diet groups, respectively, and weight decreased by 8% and 7%, respectively. The ketogenic diet group achieved greater improvements in triglyceride and HDL-cholesterol levels than the Mediterranean-style diet group but had higher LDL-cholesterol levels (percentage change +10% vs –5%, respectively). The diets were ad libitum but participants in both groups reported consuming on average 1046–1255 kJ/day (250–300 kcal/day) less compared with baseline. The ketogenic diet group had a lower fibre intake and consumed lower levels of micronutrients (folate, vitamin C and magnesium). This study was of short duration and longer term research is needed, but its findings do not justify achieving a low-carbohydrate status by avoiding fruits, legumes and whole grains, which are considered part of a healthy diet in other longstanding research.

In sum, the consideration of nutrients in isolation has led to unhelpful polarised debates on whether low-fat or low-carbohydrate diets are superior. Macronutrients are not homogeneous entities: individual nutrients are derived from foods and people eat food in overall dietary patterns.

## Beyond nutrients: foods and dietary patterns

Foods are complex mixtures of thousands of components—the food matrix—that have different physicochemical properties and health effects. This is illustrated by the opposite directions of association with the incidence of CHD seen for different foods rich in saturated fats. Consumption of dairy products such as yoghurt and cheese is inversely related to CHD incidence whereas consumption of red and processed meat is positively associated with CHD incidence [57]. This was corroborated by research showing that people who ate more saturated fats from red meat and butter were more likely to develop CHD than those who ate more saturated fats from cheese, yoghurt and fish [58]. This highlights the need to consider food sources together with the macronutrients they contain rather than the nutrients in isolation.

A consensus on dietary factors for the prevention of type 2 diabetes has been established from the comprehensive evidence base and incorporated into dietary guidelines. Broadly this suggests the benefits of the consumption of fruit, vegetables, nuts, seeds, wholegrains and yoghurt and the potential harms associated with sugar-sweetened beverages and red and processed meat. For some foods, such as fruit juice, artificially sweetened beverages, lean and fatty fish, milk and eggs, uncertainty remains with regard to their benefits for type 2 diabetes prevention [14].

Highly processed or ultra-processed foods of both plant and animal origin are increasingly consumed globally and have been related to a number of adverse health impacts. They include foods that have undergone industrial processing and that contain added ingredients such as salt, sugar, fat and artificial preservatives, stabilisers or colours, prolonging shelf life and reducing cost. An RCT compared the ad libitum consumption of ultra-processed foods with consumption of unprocessed foods. A total of 20 participants received all meals, matched for energy and macronutrient content, in a controlled setting for 28 days [59]. Ultra-processed food consumption led to substantially greater energy intake (+2090 kJ/day [+500 kcal/day] on average over 14 days) and weight gain (+0.9 kg over 14 days vs weight loss of equal magnitude during the 14 days of the unprocessed diet phase). Longer term prospective studies have provided evidence for an association of ultra-processed food consumption with the development of type 2 diabetes [60].

A number of food-based dietary patterns have a place in the prevention of type 2 diabetes based on observational evidence, including the Mediterranean, DASH and plant-based diets, but only the Mediterranean diet has been investigated in an RCT, both for the prevention and for the management of type 2 diabetes [61]. For many named popular diets such as the paleo, Atkins, Ornish and Zone diets, there is RCT evidence for short-term weight management but without any meaningful differences between them [25], while no evidence for their role in the prevention of type 2 diabetes is available.

For dietary patterns, quality matters too. For instance, plant-based diets are generally considered healthy, but not all such diets are alike. In one study, plant-based diets that were high in refined carbohydrates or were ultra-processed were associated positively with the incidence of type 2 diabetes [62].

## Embracing complexity: key messages

### Diet is a complex risk exposure

Diet is non-binary, unlike, for example, tobacco, for which zero is best. Diet is multidimensional and hierarchical in nature. Foods belong within food groups and may be consumed unprocessed (e.g. beef or pork) or processed (e.g. ham or bacon). Foods contain nutrients (e.g. meat fat or protein as macronutrients; haem iron as a micronutrient) or additives and preservatives if processed, and are part of overall dietary patterns (e.g. the Mediterranean diet with relatively low intakes of red meat or a low-carbohydrate diet regimen with relatively high intakes of meat).

The continuum of dietary exposures should be considered, as well as ‘food substitution effects’, because when more or less of one food type is consumed it impacts the consumption of other foods as part of the overall energy intake.

### Diet is hard to measure

Tools such as food frequency questionnaires or 24 h dietary recall instruments are commonly used to assess habitual dietary intakes. Despite efforts towards validating these tools and their ability to produce credible estimates of diet–disease associations, critics have called for them to be abandoned, considering them flawed because of their reliance on memory and cognition and issues of bias and measurement error [63, 64]. Suggestions for suitable alternatives are sparse, however. Emerging digital technologies—smartphone apps, cameras for food imaging and wearable devices—hold promise but are not yet of ‘research grade’, with demonstrable validity and reliability [65]. They are also not free from measurement error, nor gaming, consciously or subconsciously. A promising complementary approach is the use of objective biomarkers of dietary intakes, for instance plasma vitamin C and carotenoids as markers for fruit and vegetable intake, or plasma omega-3 fatty acids as a marker for seafood consumption [66]. However, these too have sources of random and systematic errors as well as interpretive challenges, that is, the extent to which circulating levels reflect intake compared with metabolism.

No method is perfect, but the use of validated dietary instruments with repeat measures can approximate habitual diet. Moreover, there are benefits in using a combination of methods to harness their complementary strengths and deal with relative weaknesses.

### The study design of nutritional research is challenging

The RCT design is considered the gold standard in the hierarchy of evidence-based medicine framework, but for complex behavioural exposures such as diet, unlike for pharmaceuticals, RCTs are more challenging. The bulk of the evidence base for nutrition and health has come from long-term observational prospective cohort studies. Both observational and interventional studies have relative strengths and weaknesses. Observational studies are typically limited by confounding and bias but when rigorously conducted they can yield reliable and valid results, from which causal inference can be made [14]. Dietary RCTs have several challenges. They have a specific set of limitations including a lack of blinding, lack of an appropriate control group, issues with feasibility and cost and challenges of adherence and attrition. The inability to pinpoint the specific nutritional component(s) is another challenge, such as in some of the above-cited RCTs, which could not separate out the effects of macronutrient type and energy intake. Moreover, dietary trials can vary greatly in quality, and consistency of findings and comparability are limited by the populations and endpoints included, for example healthy or diseased participants, free-living or tightly controlled conditions, and a variety of intermediate endpoints or clinical outcomes. In practice, RCTs also suffer from poor methodology and unreliable findings, as evidenced by an appraisal of nearly 21,000 RCTs [67].

Causal inference is strengthened when there is consistent evidence from different study designs. Inferring causality from observational evidence is possible by applying the Bradford Hill criteria, and Mendelian randomisation is a tool that can be applied in some situations to evaluate causal relationships [68].

No design is perfect and the evolution of improvements in all study designs—RCTs and observational studies—must continue. New concepts are emerging, such as ‘*n*-of-1’ trials and adaptive trial design, which need robust testing in the nutrition field. There is strong concordance in findings from prospective observational studies and RCTs and the two study designs should complement each other [7]. The best evidence base is that which evaluates all the relevant diverse types of evidence.

### Uncertainty remains for some dietary factors

Consensus on the potential benefits and harms of many foods and dietary patterns has been established. However, for some dietary factors controversy remains, for example in the case of non-nutritive or artificial sweeteners such as aspartame, saccharin and sucralose. These sugar substitutes can help decrease daily energy and carbohydrate intakes but whether they are helpful for obesity and type 2 diabetes in the long term is debated [69]. The use of such sweeteners is predicted to rise in line with the public health policy on sugar reduction, which in the UK includes a soft drinks industry levy applied to soft drinks containing high amounts of added sugar; manufacturers have responded to this with reformulations using sugar substitutes. To resolve this uncertainty, future research will ideally use a combination of research designs including well-conducted short-term RCTs and long-term prospective studies and employ nutritional biomarkers of artificial sweeteners.

### Noise and confusion are commonplace in the nutritional field

Everyone is interested in food. From news media to social media, books and blogs, information and misinformation on nutritional topics is everywhere. Conflicts of interest cannot always be avoided. Trusted resources are needed, including high-quality research evidence, improved dietary guidelines [70] and greater involvement of academic institutions and health agencies.

### There are many influences on what we eat beyond individual lifestyle choice (Fig. 1)

There is a gap between dietary advice and dietary intakes. Consider the public health message to eat five portions a day of fruit and vegetables. Despite strong health promotion efforts, ~12% of the population aged over 15 years in Europe meet this goal [71]. In a global context, compliance with eating five portions a day of fruit and vegetables is affected disproportionately by income, such that achieving this goal costs an estimated 52%, 18%, 16% and 2% of household income in low-, low- to middle-, middle- to upper- and high-income countries, respectively [72]. Further, sobering current examples of wider determinants of food choice include the effects of Brexit, the COVID-19 pandemic and the Russian invasion of Ukraine on availability, access and food security.

To improve and maintain dietary adherence, there is a need to operate both at the individual level and in the policy space across the entire food system (see Text box ‘Strategies to promote dietary adherence to healthy eating’). Education, dietary guidelines and strategies that enable people to make healthy food choices are necessary but not yet universally available. 
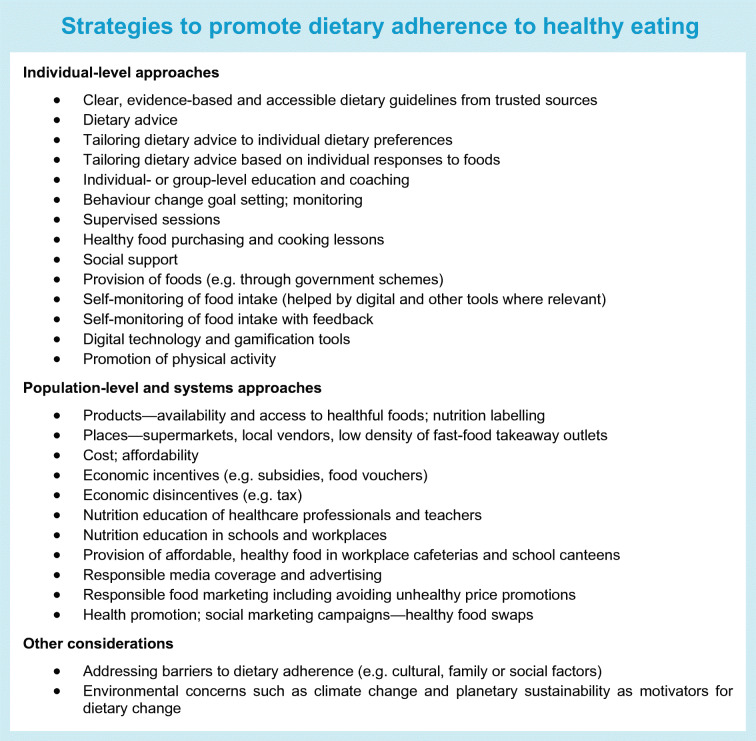

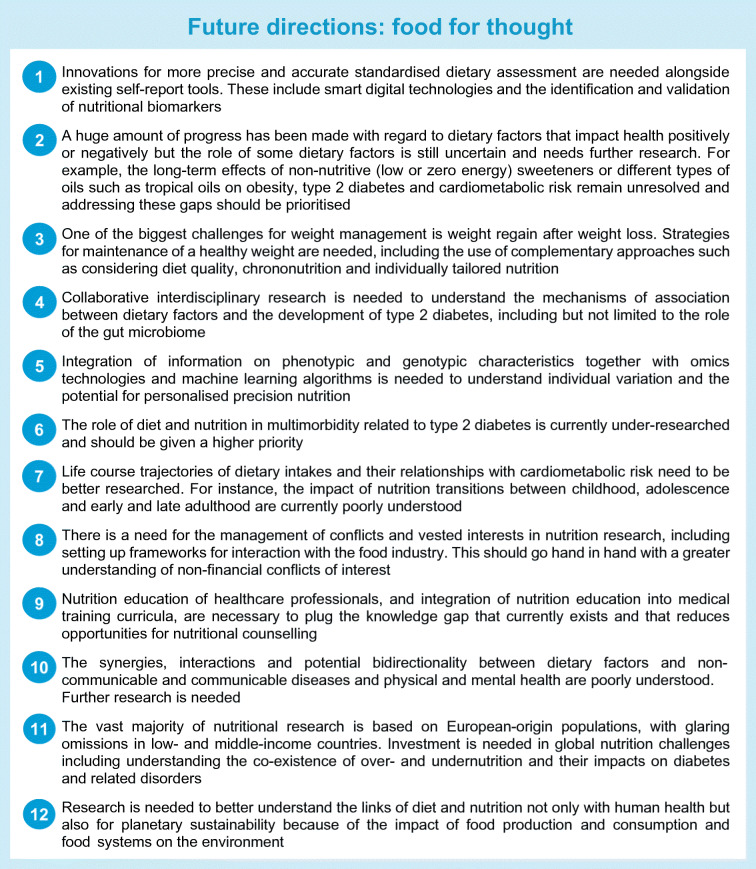


Interest has recently risen in ‘food is medicine’ interventions in healthcare systems, such that a healthy diet can be prescribed in a manner equivalent to the prescription of medication, particularly for those with food insecurity. Such interventions include food prescriptions or the provision of medically tailored groceries or meals, which in those with diabetes can achieve improvements in diet quality and in HbA_1c_ of a comparable magnitude to those seen with glucose-lowering medication [73]. Pilot data in people with uncontrolled type 2 diabetes and food insecurity are impressive, with substantial reductions in HbA_1c_ in those enrolled to receive fresh food on prescription [74]. Similarly, a meta-analysis of healthy food prescription programmes reported that an increase in consumption of fruit and vegetables by a mean of 0.8 daily servings was associated with significant reductions in BMI and HbA_1c_ [75]. Although there were methodological limitations, these studies highlight the potential effectiveness of such dietary interventions and the case for investment in further research.

### There are exciting new developments on the horizon

This is illustrated by two examples. First, greater understanding of the relationships between eating and circadian biology is emerging to shed light on so-called chrononutrition [76]. In addition to considerations of quantity and quality appraised above, chrononutrition considers the impact of the timing of food intake on metabolic health. As an example, the benefits of intermittent fasting and time-restricted feeding are becoming apparent for weight loss [77] and health more broadly [78], but research specifically targeted at type 2 diabetes is needed. Second, to improve on current dietary guidance, which is based on population averages, promising research on ‘precision nutrition’ aims to combine information from personal, biological, social and environmental factors to target individuals or population subgroups sharing similar characteristics [79]. Although still in its infancy, the use of technologies that enable information from genetics, metabolomics, proteomics and the gut microbiome to be integrated with clinical and biochemical data together with machine learning has the potential to enable the development of personalised nutrition interventions [80].

## Conclusions

Diet and nutrition play a central role in both the prevention and the management of type 2 diabetes but the complexity of diet and some key controversies have posed challenges in the field. The latest research evidence has advanced our understanding of the importance of shifting away from the decades-long focus on the quantity of isolated nutrients to nutrient quality, nutrient food sources and overall dietary patterns. New advances in research hold promise for helping to resolve current ongoing uncertainties, and exciting future directions are anticipated (see Text box ‘Future directions: food for thought’).

## Supplementary information

Figure slide (PPTX 232 kb)

## Abbreviations

DASH: Dietary Approaches to Stop Hypertension
GI: Glycaemic index
GL: Glycaemic load
NHS: National Health Service
