# Supplementary material for: Embracing complexity: making sense of diet, nutrition, obesity and type 2 diabetes
Source: Diabetologia. 2023 Feb 14;66(5):786–99. doi: 10.1007/s00125-023-05873-z (PMC9925928; doi:10.1007/s00125-023-05873-z)
Supplement: Supplementary file 1 — (PPTX 232 kb) [file 125_2023_5873_MOESM1_ESM.pptx]

## Slide 1
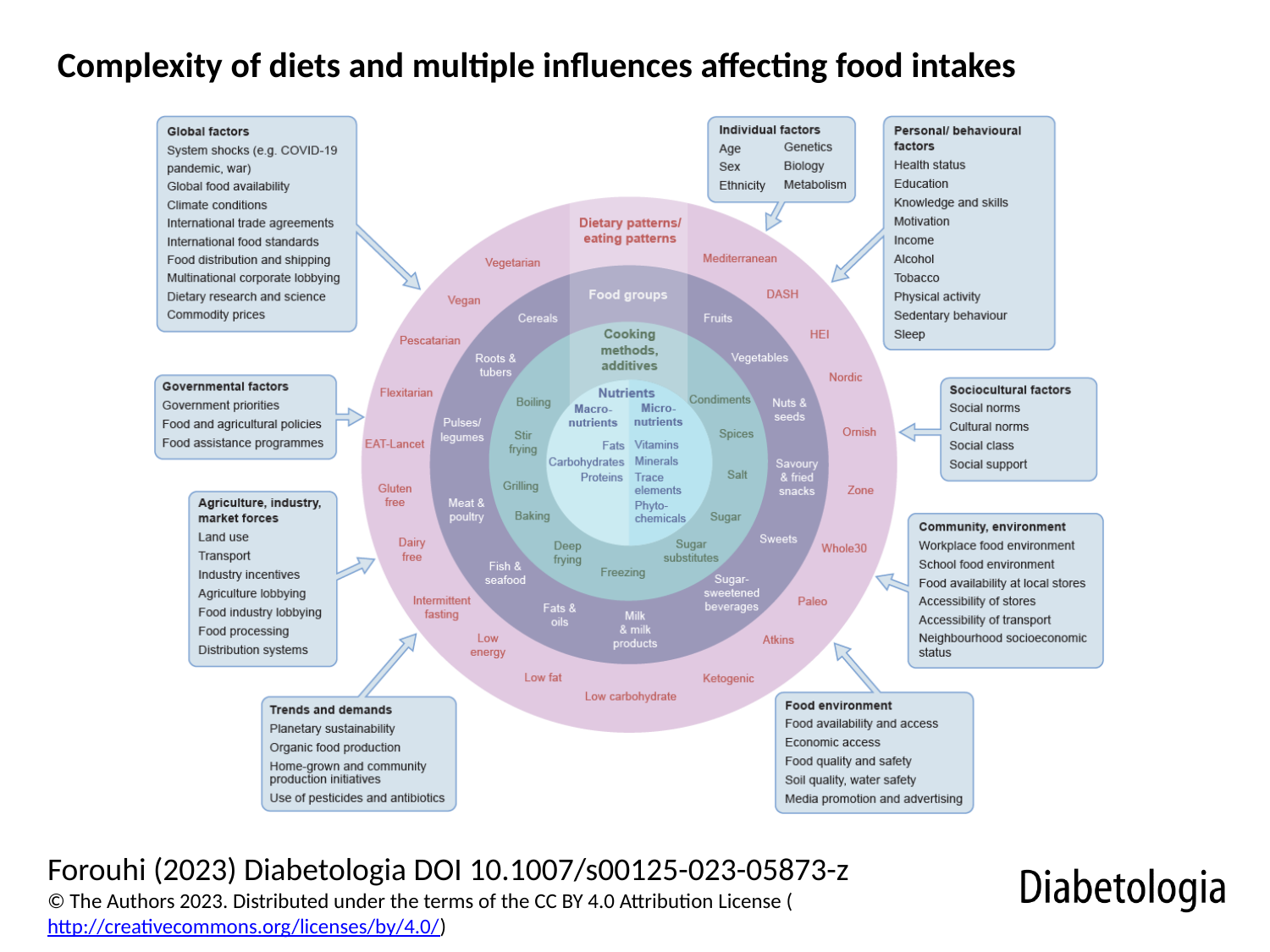

Complexity of diets and multiple influences affecting food intakes
Forouhi (2023) Diabetologia DOI 10.1007/s00125-023-05873-z
© The Authors 2023. Distributed under the terms of the CC BY 4.0 Attribution License (http://creativecommons.org/licenses/by/4.0/)
